# Identification of the RNA m5C methyltransferase genes in *Populus alba* × *Populus glandulosa* and the role of *PagTRM4B* in wood formation

**DOI:** 10.48130/forres-0025-0025

**Published:** 2025-11-07

**Authors:** Jiahuan Chen, Guoning Qi, Minyi Zhong, Zhenghao Geng, Jingran Chen, Jie Zhao, Mingjia Chen, Xiaojing Yan

**Affiliations:** 1 State Key Laboratory of Tree Genetics and Breeding, Chinese Academy of Forestry, Beijing 100091, China; 2 State Key Laboratory for Development and Utilization of Forest Food Resources, Zhejiang A&F University, Hangzhou 311300, China; 3 Provincial Key Laboratory for Non-wood Forest and Quality Control and Utilization of Its Products, Zhejiang A&F University, Hangzhou 311300, China; 4 College of Life Sciences, Nanjing Agricultural University, Nanjing 210095, China

**Keywords:** N^5^-methylcytosine (m^5^C), *PagTRM4B*, Wood formation, Xylem, *P. alba* × *P. glandulosa*

## Abstract

N^5^-methylcytosine (m^5^C) RNA methylation plays an essential role in gene regulation, yet its functions in woody plant development remain poorly understood. This study systematically identified 12 putative tRNA-specific methyltransferase 4 (TRM4) homologs (*PagTRM4s*) within the *P. alba* × *P. glandulosa* genome*.* Phylogenetic and synteny analyses revealed high evolutionary conservation across Arabidopsis and rice. Structural and domain analyses suggested functional conservation among allelic pairs. Expression profiling showed that *PagTRM4B-a* maintained stable expression in xylem at different developmental stages. *PagTRM4B-a* overexpression in poplar resulted in decreased plant height and stem diameter, modified wood composition, and increased RNA m^5^C levels. Histological analysis showed that overexpression enhanced xylem cell expansion and secondary xylem formation without affecting cambial activity. Moreover, the expression levels of secondary cell wall (SCW) biosynthesis genes were significantly down-regulated in *PagTRM4B-a-OE* transgenic plants. This study offers new insights into the epigenetic control of secondary growth in trees and highlights *PagTRM4B-a* as a potential target for influencing wood formation in woody plants.

## Introduction

Wood formation, or xylogenesis, is a distinct process of secondary growth in perennial woody plants, characterized by coordinated biological events, such as periodic cambial cell division, xylem precursor cell differentiation, secondary cell wall deposition, and programmed cell death^[1,2]^. This process is crucial for determining the mechanical support and transport efficiency of vascular plants, as well as playing a key role in carbon fixation and biomass accumulation^[3]^. Wood formation is governed by a complex regulatory network that integrates various control layers at the molecular level. Transcriptional regulators, including NAC, MYB, and HD-ZIP III family transcription factors, play crucial roles in controlling developmental transitions from cambial activity to xylem differentiation and secondary cell wall biosynthesis^[4−8]^. Phytohormone signaling pathways, including auxin, ethylene, gibberellins, and jasmonic acid, are essential for coordinating the spatial and temporal aspects of secondary growth^[9−15]^. Epigenetic mechanisms, including histone methylation and acetylation, play roles in dynamically regulating gene expression during wood development^[16−18]^. However, additional regulatory factors and pathways involved in wood formation are still needed, as most of the currently identified components control only a limited subset of target genes. Identifying some missing elements is essential for constructing a more comprehensive model of wood formation at the molecular level.

RNA post-transcriptional modifications are prevalent across living organisms and play a crucial role in regulating gene expression^[19,20]^. N^6^-methyladenosine (m^6^A) is the most common internal modification in eukaryotic mRNAs, playing a role in numerous biological processes, such as heat shock stress^[21]^, arsenite-induced oxidative stress^[22,23]^, root meristem cell proliferation, tiller bud formation, photosynthetic efficiency, and drought tolerance^[24]^. Another abundant internal RNA modification is 5-methylcytosine (m^5^C), which has been detected at thousands of sites in humans, Arabidopsis, and rice mRNAs^[25,26]^. m^5^C deposition is facilitated by conserved methyltransferases from the NOP2/Sun (NSUN) domain protein family, such as NSUN2 in mammals, tRNA-specific methyltransferase 4B (TRM4B) in *Arabidopsis*, and OsNSUN2 in *Oryza sativa*^[27−29]^. Recent research indicates that mRNA m^5^C is crucial for root development processes and abiotic stress tolerance in plants. OsNSUN2-mediated m^5^C in rice improves adaptation to elevated temperatures^[29]^. Consistently, the knockout of *OsNSUN2* results in shorter root length, indicating its role in root development as well^[29]^. In Arabidopsis, the *trm4b* mutant, deficient in a crucial m^5^C methyltransferase, shows heightened oxidative stress sensitivity, along with shorter primary roots and fewer lateral roots^[30,31]^. Root growth defects correlate with reduced cell division in the root apical meristem, indicating that TRM4B-mediated m^5^C is crucial for sustaining meristem function and root architecture^[32]^. Despite increasing interest in m^5^C RNA modifications in model plants like Arabidopsis and rice, their functions in the secondary growth of perennial woody species are still not well understood. This study systematically analyzed the gene family composition, phylogenetic relationships, and expression patterns of the m^5^C methyltransferase TRM4 in *P. alba* × *P. glandulosa* and investigated the potential functional mechanisms of *PagTRM4B* in wood formation.

## Materials and methods

### Identification and phylogenetic analyses of *PagTRM4s*

Nucleotide and protein sequences of the TRM4 family from *A. thaliana*, *Oryza sativa, P. trichocarpa*, and *Homo sapiens* were obtained from the Phytozome database (https://phytozome-next.jgi.doe.gov). The genome data for *P. alba* × *P. glandulosa* were sourced from the Figshare database (doi: 10.6084/m9.figshare.12369209). TRM4 family members in *P. alba* × *P. glandulosa* were identified through BLAST analysis using TRM4 protein sequences from *P. trichocarpa* and *A. thaliana* as queries, applying an *E*-value cutoff of 1e-10.

For phylogenetic analysis, TRM4 protein sequences were aligned using ClustalW and trimmed to remove poorly aligned regions. A phylogenetic tree was constructed in MEGA12.0 using the Neighbor-Joining (NJ) method, with 1,000 bootstrap replicates, to evaluate branch reliability. The phylogenetic tree was visualized via the Interactive Tree of Life (iTOL) platform (https://itol.embl.de/).

The ExPASy ProtParam tool (http://web.expasy.org/protparam/) was utilized to analyze the physicochemical properties of the identified PagTRM4 proteins, such as amino acid length, molecular weight, isoelectric point (pI), hydrophobicity/hydrophilicity, and stability index.

### Chromosomal distribution and synteny analysis of *PagTRM4* genes

*PagTRM4* gene locations in the *P. alba* × *P. glandulosa* genome were identified using GFF annotation files, and visualized with TBtool's Gene Location Visualize module. Synteny analysis using MCScanX was conducted to examine the evolutionary conservation of TRM4A genes, identifying collinear gene pairs between *P. trichocarpa* and *P. alba* × *P. glandulosa*, as well as between *P. alba* × *P. glandulosa*, and *A. thaliana*. Graphical representations of the results were created using the circlize and ComplexHeatmap packages in R.

### Gene structure and motif analyses

MEME was utilized to identify conserved domains and motifs in PagTRM4 proteins, with the motif limit set to 10, and default settings for other parameters. Gene structures, encompassing intron-exon arrangement and untranslated regions (UTRs), were annotated using GFF3 data from the *P. alba* × *P. glandulosa* genome database. TBtools was used to visualize all results.

### Materials and conditions for plant growth

*P. alba* × *P. glandulosa* plants were grown in a greenhouse with peat moss substrate under long-day conditions (16 h light/8 h dark), at a consistent temperature of 25 °C during the day, and 23 °C at night. Sterile *P. alba* × *P. glandulosa* plantlets were propagated via stem cuttings on 1/2 MS medium (pH 5.8–6.0), with 30 g/L sucrose, 5.8 g/L agar, 0.1 mg/L indole-3-butyric acid (IBA), and 0.01 mg/L *α*-naphthylacetic acid (NAA). Xylem from 20-day-old and 6-month-old greenhouse plants were collected and preserved in liquid nitrogen for RNA extraction. Xylem samples from 10-year-old *P. alba* × *P. glandulosa* trees were collected at Beiwu garden (39°59' N, 116°15' E; Beijing, China), and preserved in liquid nitrogen for subsequent analyses.

### Vector construction and plant transformation

The *PagTRM4B-a* coding region was PCR-amplified from *P. alba* × *P. glandulosa* xylem cDNA and cloned into the pBI121-2 × 35S-3 × Flag vector using Exnase II-mediated homologous recombination with the ClonExpressII One Step Cloning Kit (Vazyme Biotech Co., Ltd, Nanjing, Jiangsu, China), creating the expression construct pBI121-2 × 35S-3 × Flag:PagTRM4B-a. The resulting plasmid was introduced into *Agrobacterium tumefaciens* strain GV3101 by electroporation. Young leaf discs served as explants for the genetic transformation of *P. alba* × *P. glandulosa*. Following Agrobacterium-mediated transformation, explants were co-cultivated on callus induction medium and subjected to selection under appropriate antibiotic pressure to generate overexpressing transgenic lines (*35S:PagTRM4B*). Positive transformants were screened by PCR at the genomic DNA level, and expression levels were subsequently confirmed by RT-qPCR analysis. Supplementary Table S1 lists the primers used for vector construction and identification.

### Growth and histological phenotypic analyses

Measurements of plant height, stem diameter, and stem internode count were taken from 5-month-old *P. alba* × *P. glandulosa* plants using a standard measuring tape and vernier caliper. For histological analysis, stem segments from the 7^th^ internode were excised and fixed in place using Loctite 495 adhesive then transversed and sectioned into 50 µm using a LeicaVT1000s oscillating microtome (Leica, Wetzlar, Germany). Sections were stained with 0.05% toluidine blue for 1 min, and examined using an OLYMPUS BX51 microscope (Olympus Corp., Tokyo, Japan).

### Quantitative analyses of the m^5^C/C ratio in RNA by LC-MS/MS

mRNA was extracted from total RNA using the PolyATtract® mRNA Isolation System IV (Promega, Madison, Wl, USA) following the manufacturer's guidelines. Both mRNA and total RNA samples were then fully digested into single nucleosides, as previously described^[25,33,34]^. Each sample (1 μg) was digested in a 50 μL buffer with 10 mM Tris–HCl (pH 7.9), 1 mM MgCl_2_, 0.1 mg/mL BSA, 0.4 units benzonase (Sigma-Aldrich, St. Louis, MO, USA), 0.004 units phosphodiesterase I (Sigma-Aldrich, St. Louis, MO, USA), and 0.04 units shrimp alkaline phosphatase (NEB, Beverly, MA, USA). Following a 10-h incubation at 37 °C, the enzymatic reaction was halted, and samples were filtered through a 3 kDa cutoff ultrafiltration tube. 2 μL aliquots were subsequently analyzed using an Agilent 1290 HPLC system coupled with a Sciex 6500 Qtrap mass spectrometer. The mass transitions monitored were m/z 244.3 to 111.9 for Cytidine (C), and m/z 258.4 to 108.8 for 5-methylcytidine (m^5^C). Standard solutions for quantification included C at concentrations of 1, 5, 25, 50, 100, 200, 400, 2,000, and 10,000 ng/mL and m^5^C at concentrations of 0.1, 0.5, 2.5, 5, 10, 20, 40, 200, and 1,000 ng/mL. The m^5^C to C ratio was determined using the calibrated concentrations.

### Analysis and quantification of lignin, cellulose, and hemicellulose contents

Lignin, cellulose, and hemicellulose levels in poplar secondary tissues were measured using kits following the provided instructions (BOXBIO, Beijing, China). Lignin content was assessed utilizing the Lignin Content Assay Kit (Cat. No. AKSU010U). This method involves acetylating the phenolic hydroxyl groups in lignin to produce acetylated lignin, characterized by an absorbance at 280 nm. Lignin content was determined by measuring absorbance with a microplate reader and referencing a standard curve of known lignin concentrations. The Cellulose (CLL) Content Assay Kit (Cat. No. AKSU007M) was utilized to measure cellulose content. The sample's cellulose was hydrolyzed to *β*-D-glucose under acidic conditions and then dehydrated in a strong acid to produce furfural. The furfural then reacted with anthrone to produce a blue-green chromogenic compound with a specific absorption peak at 620 nm. Absorbance was recorded and cellulose content was calculated using the standard curve method. The Hemicellulose Content Assay Kit (Cat. No. AKSU008C) was utilized to quantify hemicellulose content. After acid hydrolysis, hemicellulose was transformed into reducing sugars that reacted with 3,5-dinitrosalicylic acid (DNS) to produce brown-red amino compounds. These compounds exhibited a characteristic absorbance at 540 nm. Hemicellulose concentration was determined by using absorbance measurements compared to a standard curve.

### RNA extraction and RT-qPCR analysis

The CTAB method was employed to extract total RNA from poplar tissues^[35]^. RNA quality and concentration were assessed by NanoDrop and agarose gel electrophoresis. RNA (1 µg) was converted to cDNA using the HiFiScript gDNA Removal cDNA Synthesis Kit (CWBIO, Taizhou, China). SYBR Green based RT-qPCR was conducted using the Roche LightCycler 480 system (Roche, Basel, Switzerland). Each 20 μL reaction comprised 2 μL of cDNA, 10 μL of SYBR Master Mix, 0.4 μL of each 10 μM primer, and 7.2 μL of nuclease-free water. The cycling protocol involved an initial step at 95  °C for 10 min, followed by 40 cycles of 95  °C for 10 s, and 60  °C for 30 s. Each sample underwent analysis with three biological, and three technical replicates. Relative expression levels were determined using the 2^−ΔΔCᴛ^ method, employing *PagActin* (*Potri.001G309500*) as the reference gene. Refer to Supplementary Table S1 for the list of primers.

## Results

### Identification and phylogenetic analyses of *TRM4* genes in *P. alba* × *P. glandulosa*

**Figure 1:**
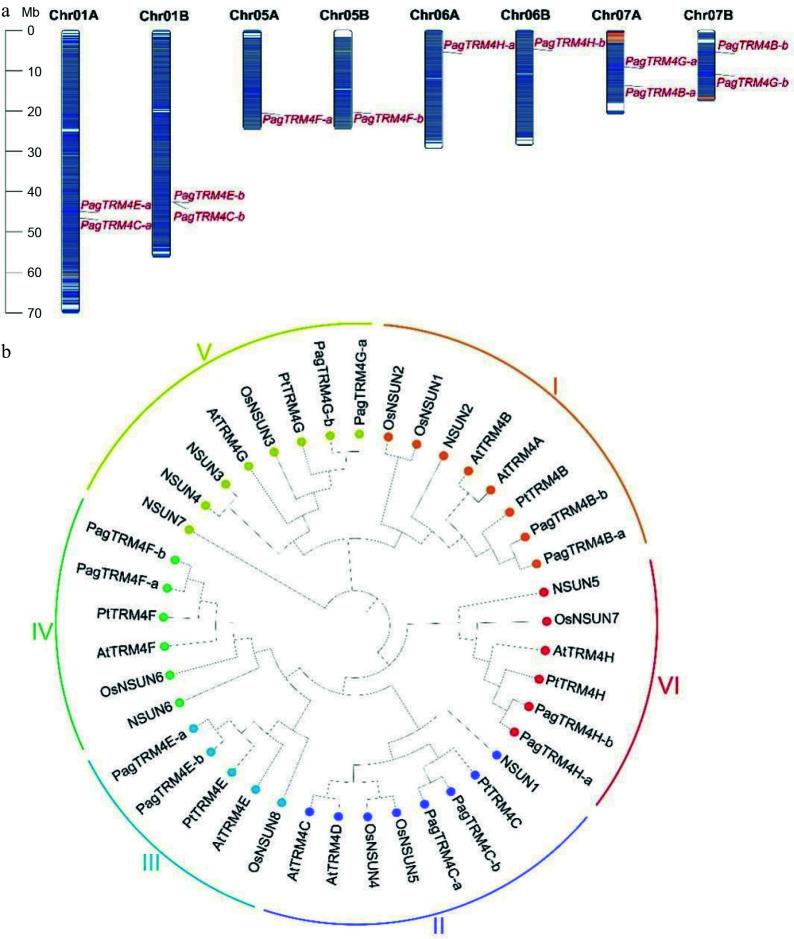
Chromosome distribution and phylogenetic analysis of *PagTRM4* genes in *P. alba* × *P. glandulosa*. (a) Chromosomal localization of *PagTRM4* genes across the eight chromosomes of the A and B subgenomes in *P. alba* × *P. glandulosa*. (b) Phylogenetic tree of TRM4 proteins from *Homo sapiens*, *A. thaliana*, *Oryza sativa*, *P. trichocarpa,* and *P. alba* × *P. glandulosa*. The tree was constructed using the ClustalW alignment and Neighbor-Joining (NJ) method with 1,000 bootstrap replicates in MEGA 12.0. Based on sequence homology, TRM4 proteins were classified into six distinct subfamilies (I to VI), indicated by colored circles and arcs.

The *TRM4* gene family in the *P. alba* × *P. glandulosa* genome (v3.1) was systematically identified using a combination of Hidden Markov Models (HMMs) and BLASTp analyses (version 2.16.0). An HMM-based search was conducted using the conserved TRM domain (PF01066) as the query. After manual curation and removal of redundant or non-conserved sequences, 12 putative *PagTRM4* genes were identified (Supplementary Table S2). Chromosomal localization analysis revealed a symmetric distribution of these genes across the genome, with members mapped to eight different chromosomes in both the A and B subgenomes. Based on their phylogenetic relationship to Arabidopsis *TRM4* genes and their respective chromosomal positions, these genes were designated as *PagTRM4A-a* (*PagTRM4A-b*) through *PagTRM4G-a* (*PagTRM4G-b*) (Fig. 1a).

A comparative phylogenetic analysis was performed to investigate the evolutionary dynamics of *PagTRM4* genes, utilizing seven NSUN protein sequences from *Homo sapiens*, eight AtTRM4 protein sequences from *A. thaliana*, eight OsNSUN protein sequences from *Oryza sativa*, six PtTRM4 protein sequences from *P. trichocarpa*, and 12 PagTRM4 protein sequences from *P. alba* × *P. glandulosa*. The phylogenetic tree was categorized into six evolutionary clades according to the Arabidopsis subfamily classification, with support from sequence conservation patterns and domain architecture (Fig. 1b). A synteny analysis was performed to examine the evolutionary conservation of *TRM4* genes by identifying interspecies collinearity between *P. alba* × *P. glandulosa* and two other species. The analysis identified 10 collinear *TRM4* gene pairs between *P. alba* × *P. glandulosa* and *A. thaliana*, and another 10 collinear pairs between *P. trichocarpa* and *P. alba* × *P. glandulosa* (Supplementary Fig. S1, Supplementary Tables S3 & S4). These results suggest a high degree of evolutionary conservation of the *TRM4* gene family across these species.

### Analysis of *PagTRM4* genes focused on their gene structure and conserved motifs

**Figure 2:**
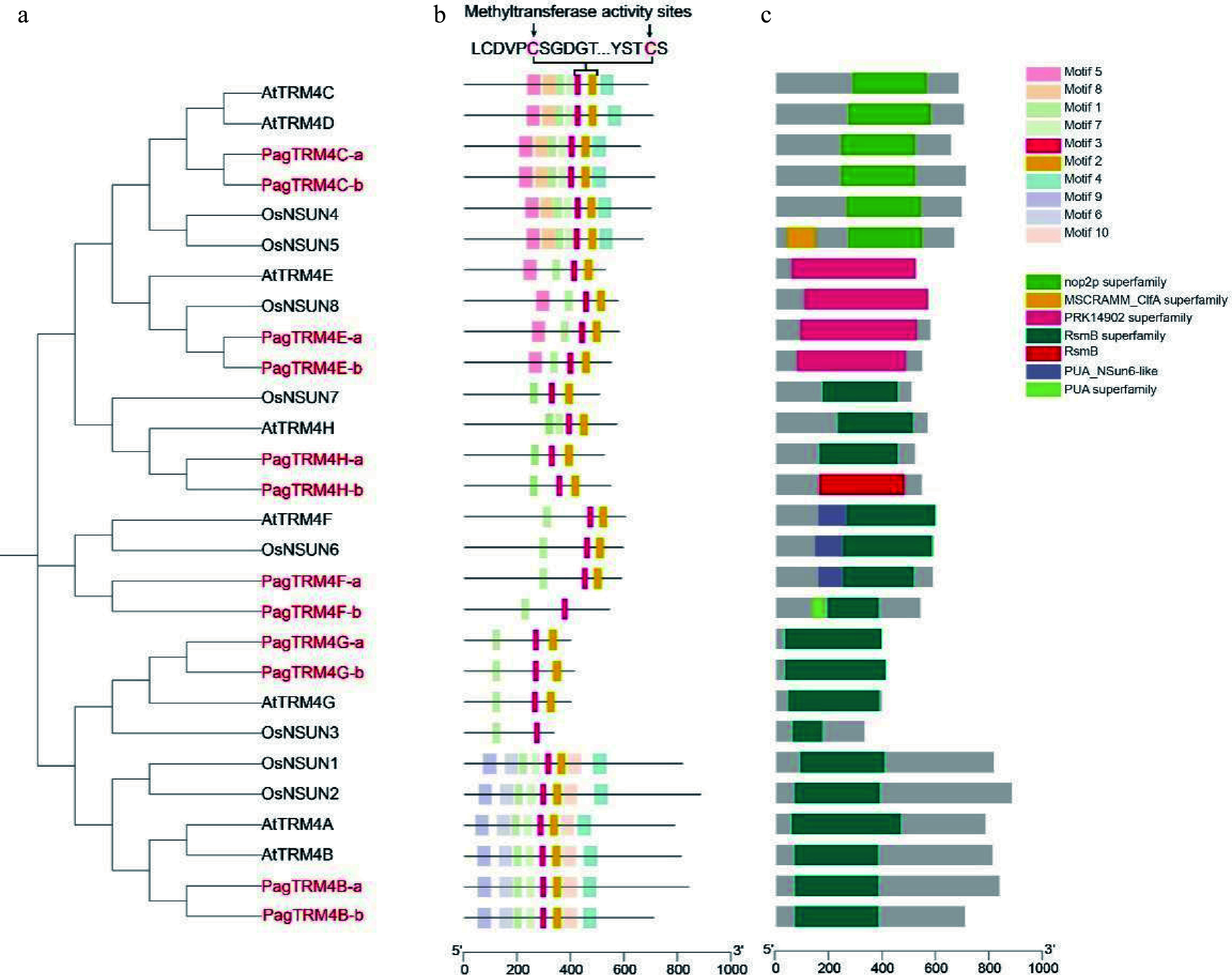
Phylogenetic analysis and conserved features of TRM4 family protein genes in three plant species. (a) Phylogenetic relationships of TRM4 proteins from *A. thaliana*, *Oryza sativa,* and *P. alba* × *P. glandulosa*. (b) Conserved motifs within TRM4 proteins were identified using the MEME tool (version 5.5.7), resulting in the identification of 10 unique motifs, designated as Motif 1 through Motif 10. (c) The schematic representation of conserved domains illustrates their positions and sizes, with each domain depicted as a colored square.

To further characterize the 12 *PagTRM4* genes, gene structure and conserved motif analyses was performed. The study found that while most allelic gene pairs exhibited similar exon-intron structures, notable variations were present in the 5' and 3' untranslated regions (UTRs) of specific alleles, including *PagTRM4E*-*a*/*b* and *PagTRM4G*-*a*/*b* (Supplementary Fig. S2). Ten conserved motifs (Motifs 1−10) were identified, with Motifs 2 and 3 being the most conserved, containing the crucial cysteine residues necessary for methyltransferase activity (Fig. 2a, b; Supplementary Fig. S3). The survey conducted domain analysis further confirmed that the TRM4 proteins in poplar share similar domain compositions with their homologs in Arabidopsis and rice. For example, PagTRM4C-a/b contains the Nop2p superfamily domain, PagTRM4E-a/b possesses the PRK14902 superfamily domain, and PagTRM4H/F/G/B-a/b harbor the RsmB superfamily domain (Fig. 2c). Phylogenetic analysis showed that genes clustered within the same clade also shared conserved gene structures and domain features, suggesting that they may have undergone similar evolutionary trajectories.

### Tissue-specific expression patterns of *PagTRM4* genes

**Figure 3:**
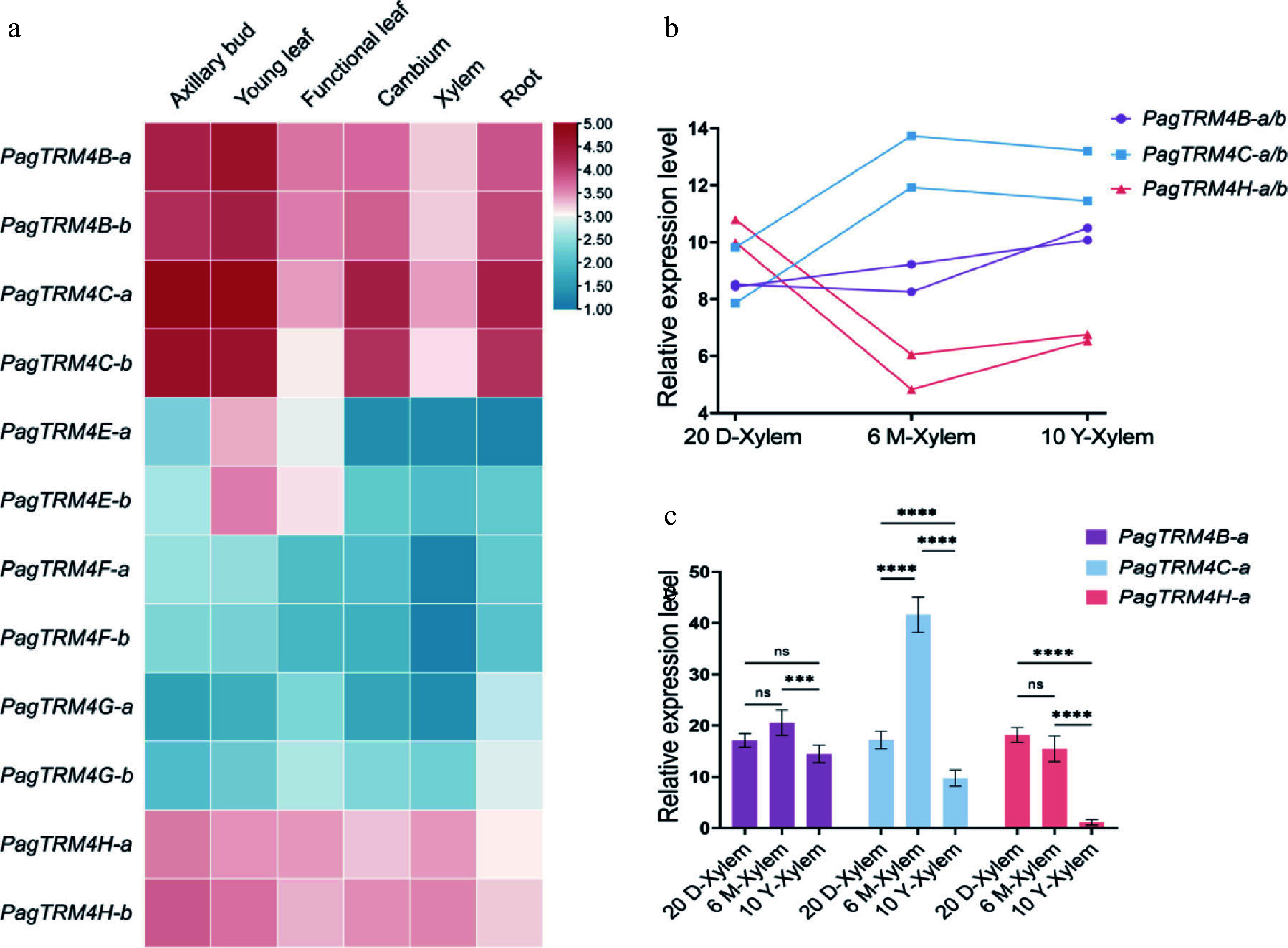
Expression profiles of *PagTRM4* genes in *P. alba* × *P. glandulosa* in 20-day-old (20 D), 6-month-old (6 M), and 10-year-old (10 Y) trees. (a) Heatmap displaying the transcript abundance of *PagTRM4* genes across different tissues, including axillary buds, young leaves, functional leaves, cambium, xylem, and roots. The color scale (blue to red) represents low to high expression levels. (b) Expression patterns of *PagTRM4B*/*C*/*H*-*a*/*b* genes in the xylem of different developmental stages. (c) RT-qPCR analysis of *PagTRM4B*/*C*/*H*-*a* genes in xylem tissues collected from 20-day-old (20 D-Xylem), 6-month-old (6 M-Xylem), and 10-year-old (10 Y-Xylem) trees. Error bars represent mean with SD. Asterisks indicate statistically significant differences among samples (two-way ANOVA with Tukey's post-hoc test for pairwise comparisons; *** *p* < 0.001; **** *p* < 0.0001; n = 3 biological replicates, each with three technical replicates).

*PagTRM4* gene expression patterns were examined using published RNA-seq data from six *P. alba* × *P. glandulosa* tissues: axillary bud, young leaf, functional leaf, cambium, xylem, and root^[36]^. The findings indicated that *PagTRM4B*-*a*/*b*, *PagTRM4C*-*a*/*b*, and *PagTRM4H*-*a*/*b* were ubiquitously expressed in all tissues, with notably higher expression levels in axillary bud, young leaf, cambium, and root. *PagTRM4E*-*a*/*b* expression was detected exclusively in young and functional leaves, whereas *PagTRM4F*-*a*/*b* and *PagTRM4FG*-*a*/*b* exhibited very low expression levels across all tissues (Fig. 3a). Notably, *PagTRM4B*-*a*/*b*, *PagTRM4C*-*a*/*b*, and *PagTRM4H*-*a*/*b* were expressed in tissues associated with secondary growth, such as the cambium and xylem. The expression patterns of *PagTRM4B*-*a*/*b*, *PagTRM4C*-*a*/*b*, and *PagTRM4H*-*a*/*b* genes in the xylem of 20-day-old, 6-month-old, and 10-year-old trees were examined using existing transcriptome data to clarify their roles in wood formation^[36−38]^. The results showed that *PagTRM4B*-*a*/*b* maintained relatively stable expression levels across all three developmental stages. *PagTRM4C*-*a*/*b* showed low expression in the xylem of 20-day-old trees, but its expression was significantly higher in the xylem of 6-month-old, and 10-year-old trees. *PagTRM4H*-*a*/*b* exhibited peak expression in the xylem of 20-day-old trees, followed by a significant decline in expression as development progress (Fig. 3b). RT-qPCR analysis was used to validate the expression pattern of *PagTRM4B*/*C*/*H*-*a* in xylem from trees of different ages (20 d, 6 months, and 10 years). The findings indicated that *PagTRM4B*-*a* expression was consistent in the xylem of 20-day-old, 6-month-old, and 10-year-old trees. *PagTRM4C*-*a* exhibited peak expression in 6-month-old trees, while *PagTRM4H*-*a* showed elevated expression in both 20-day-old and 6-month-old trees (Fig. 3c). These patterns suggest distinct temporal roles for the three genes during xylem development, and *PagTRM4B* may play a sustained role throughout different stages of wood development.

### Overexpression of *PagTRM4B-a* genes reduces RNA m^5^C modification levels, impacting poplar growth and development

Gene expression pattern analysis revealed that *PagTRM4B*-*a* is expressed at a relatively stable level during wood formation. To investigate PagTRM4B-a's function in secondary cell wall (SCW) formation, *PagTRM4B-a* overexpression (*PagTRM4B-a-OE*) transgenic lines were developed utilizing the CaMV 35S promoter. A total of nine independent *PagTRM4B-a-OE* transgenic lines were confirmed by PCR at the DNA level (Supplementary Fig. S4a). Further expression analysis showed that all seven lines exhibited significantly elevated *PagTRM4B-a* transcript levels in the developing xylem compared to wild-type (WT) plants (Supplementary Fig. S4b). OE-2, OE-7, and OE-9, exhibiting the highest expression levels, were chosen for further functional analyses.

**Figure 4:**
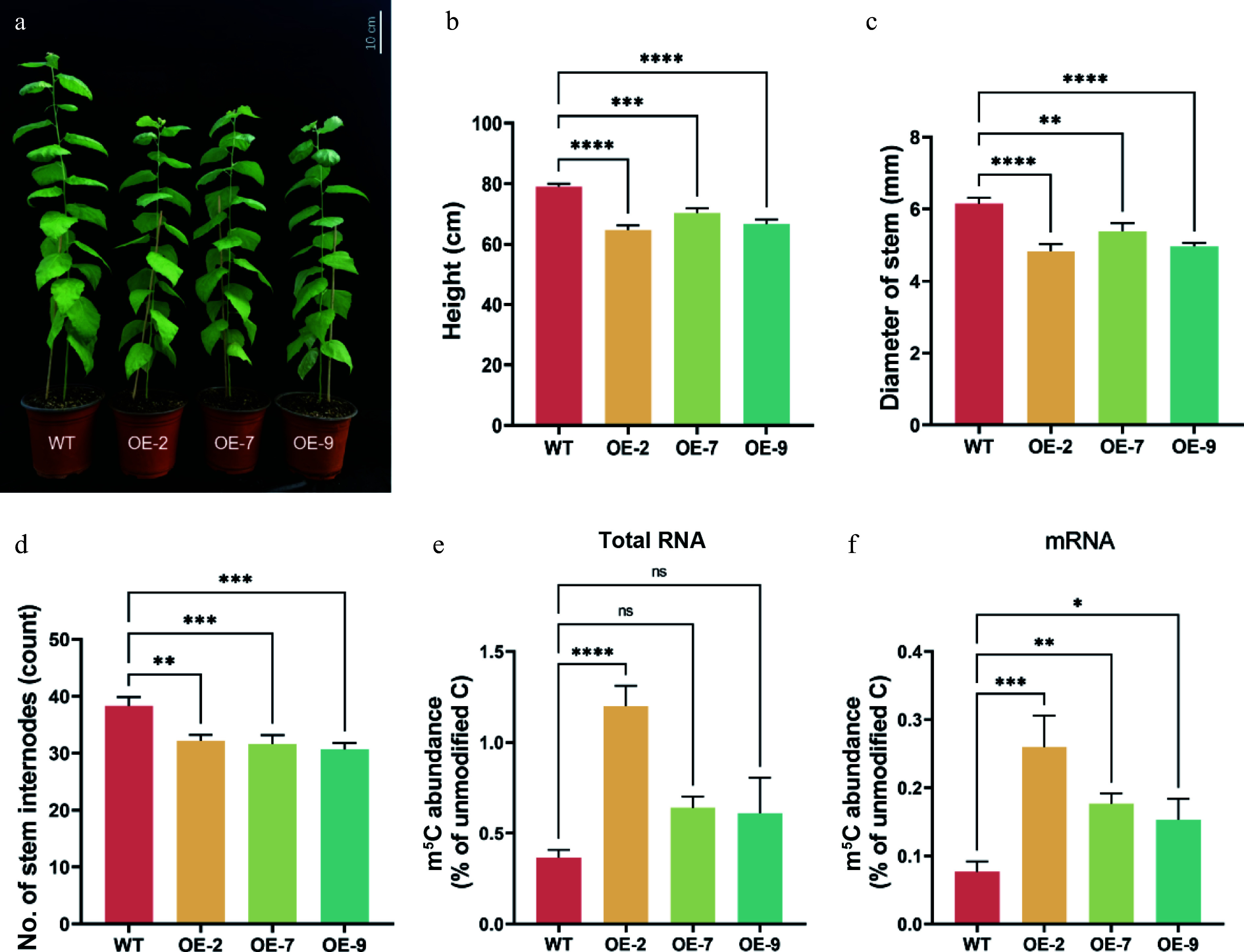
*PagTRM4B-a-OE* transgenic poplar phenotypes. (a) Five-month-old wild type and three *PagTRM4B-a* overexpression transgenic lines (OE-2, OE-7, and OE-9). Bar = 10 cm. (b)–(d) Measurement of plant height, basal stem diameter, and internode number. (e) and (f) Quantification of the m^5^C/C of total RNA and mRNA isolated from 5-month-old WT, OE-2, OE-7, and OE-9 transgenic poplar xylem by LC-MS/MS. Error bars represent mean with SD. Asterisks indicate statistically significant differences among samples (one-way ANOVA followed by Dunnett's test for pairwise comparisons; * *p* < 0.05; ** *p* < 0.01; *** *p* < 0.001; **** *p* < 0.0001; n = 3 biological replicates, each with three technical replicates).

Through phenotypic observation, it was found that, compared to WT plants, *PagTRM4B-a-OE* transgenic lines exhibited a reduced growth rate (Fig. 4a). These differences were quantified by measuring plant height, basal stem diameter, and internode number in 5-month-old plants. The findings indicated that *PagTRM4B-a-OE* lines exhibited notably reduced plant height, with OE-2, OE-7 and OE-9 showing decreases of 18.1%, 11%, and 15.6%, respectively (Fig. 4b). Basal stem diameter was also significantly reduced, by 21.4%, 12.5%, and 19.4% in the same lines (Fig. 4c). The internode number significantly decreased by 16.1%, 17.4%, and 20% in OE-2, OE-7, and OE-9, respectively (Fig. 4d). These results suggest that overexpression of *PagTRM4B-a* negatively affects multiple aspects of vegetative growth in poplar. m^5^C modification levels were evaluated in total RNA and mRNA from stem tissues of both WT and *PagTRM4B-a-OE* transgenic lines. The OE-2 showed a marked increase in m^5^C levels in both total RNA and mRNA compared to WT. While OE-7 and OE-9 showed no notable change in overall RNA m^5^C levels, their mRNA m^5^C levels were significantly elevated (Fig. 4e & f). These results suggest that *PagTRM4B-a* overexpression may influence RNA methylation in a transcript-specific manner.

### PagTRM4B-a is required for xylem cell expansion and SCW biosynthesis, but is not involved in cambial activity

**Figure 5:**
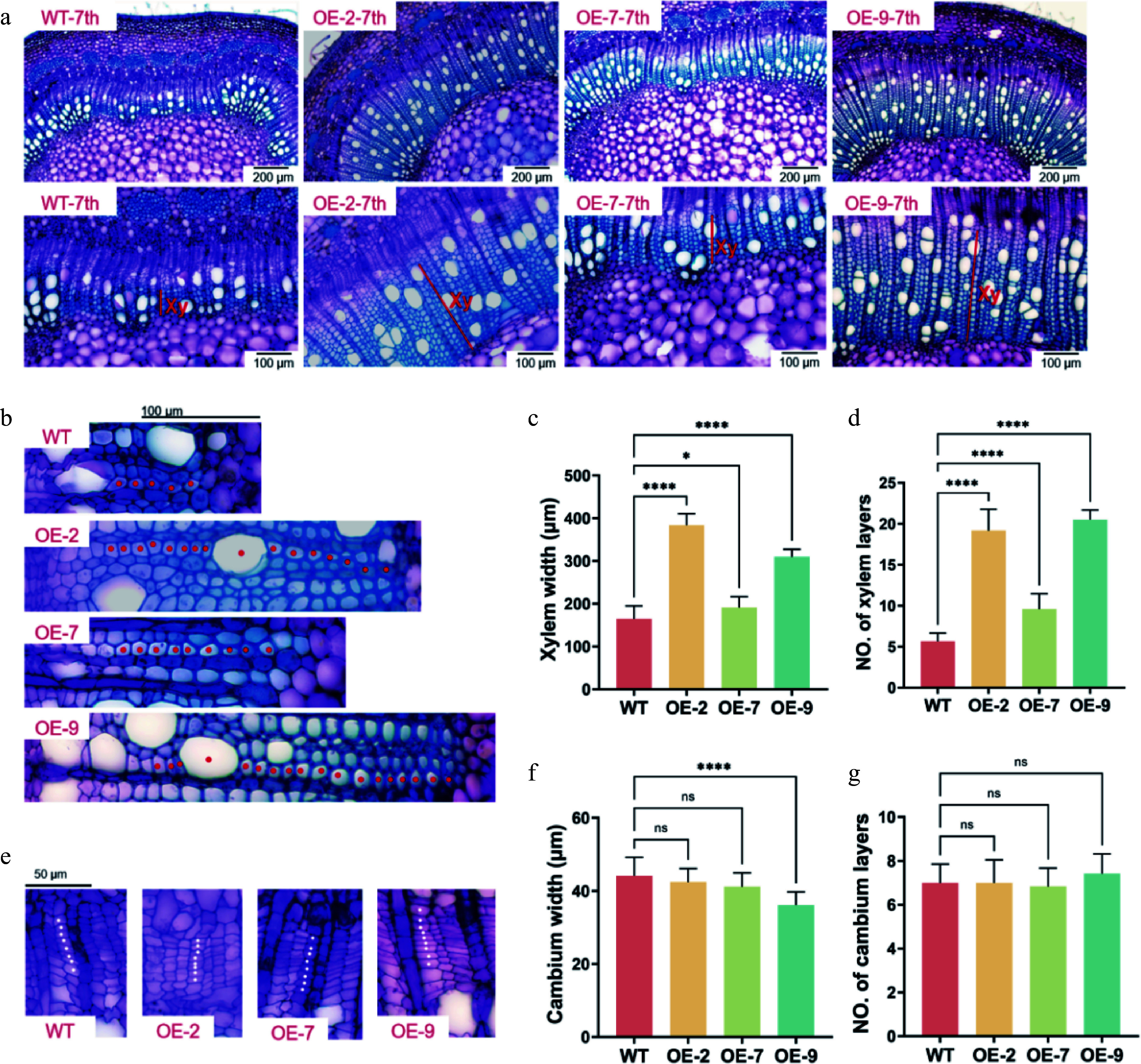
Characterization of secondary xylem development in WT and *PagTRM4B-a-OE* transgenic lines. (a) Stem cross-sections of the 7^th^ internode of 5-month-old WT, OE-2, OE-7, and OE-9, stained with toluidine blue O (TBO). Xylem width is indicated with red bars. Bars are 200 μm (top line), and 100 μm (bottom line). Xy, xylem. (b) Enlarged images in the secondary xylem region of WT, OE-2, OE-7, and OE-9. Red dots indicate vessels and fiber cells. Bars are 100 μm. (c), (d) Quantification of xylem width and xylem layers in poplar stems. (e) Enlarged images in the cambial region of WT, OE-2, OE-7, and OE-9. White dots indicate cambial cells. Bars are 50 μm. (f), (g) Quantification of cambium width and cambium layers in poplar stems. Error bars represent mean with SD. Asterisks indicate statistically significant differences between the WT and OE lines (one-way ANOVA followed by Dunnett's test for pairwise comparisons; * *p* < 0.05; **** *p* < 0.0001; n = 3 biological replicates, each with 12 technical replicates).

To further investigate the role of *PagTRM4B-a* in wood formation, the secondary growth structures of poplar stems were investigated. Stem cross-sections at the 7^th^ internode, an area of active secondary growth, were examined in 5-month-old WT and transgenic plants. The *PagTRM4B-a-OE* transgenic lines showed a significant increase in secondary xylem compared to the WT. Specifically, the xylem width increased by 132.7%, 16.1%, and 88% in the OE-2, OE-7, and OE-9 lines, respectively (Fig. 5a & c). The number of xylem cell layers increased by 238.3%，69.1%, and 261.8% (Fig. 5b & d), respectively. To determine whether this increase in xylem cell layers was associated with altered cambial activity, the cambial zones were compared between WT and transgenic lines. The findings indicated no notable variations in either the width or the number of cell layers within the cambial region (Fig. 5e−g). In addition, the difference in secondary cell wall biosynthesis between WT and *PagTRM4B-a-OE* transgenic plants were analyzed. Analysis of wood composition indicated variations in cellulose, lignin, and hemicellulose levels. The *PagTRM4B-a-OE* lines exhibited a significant increase in lignin content, while cellulose and hemicellulose contents were reduced compared to WT (Table 1). These findings suggest that *PagTRM4B-a* promotes xylem development during secondary growth, potentially by enhancing xylem differentiation and lignin accumulation during wood formation rather than cambial cell division.

**Table 1:** Wood composition in wild-type (WT), and *PagTRM4B-a-OE* (OE) transgenic plants.

Wood composition	WT	OE-2	OE-7	OE-9
Lignin	26.23% ± 0.56%	33.41% ± 1.16%***	28.49% ± 1.22%	29.86% ± 1.33%*
Cellulose	51.26% ± 1.68%	28.90% ± 0.83%****	33.58% ± 1.36%****	30.57% ± 1.82%****
Hemicellulose	18.30% ± 0.50%	15.42% ± 0.66%**	17.59% ± 0.87%	17.55% ± 0.82%
Wood compositions were analyzed using samples from 5-month-old trees. Values represent mean ± SD (n = 3). Asterisks indicate statistically significant differences among samples (one-way ANOVA followed by Dunnett's test for pairwise comparisons; * *p* < 0.05; ** *p* < 0.01; *** *p* < 0.001; **** *p* < 0.0001; n = 3 biological replicates, each with three technical replicates).				

### PagTRM4B-a is required for the expression of key marker genes during wood formation

**Figure 6:**
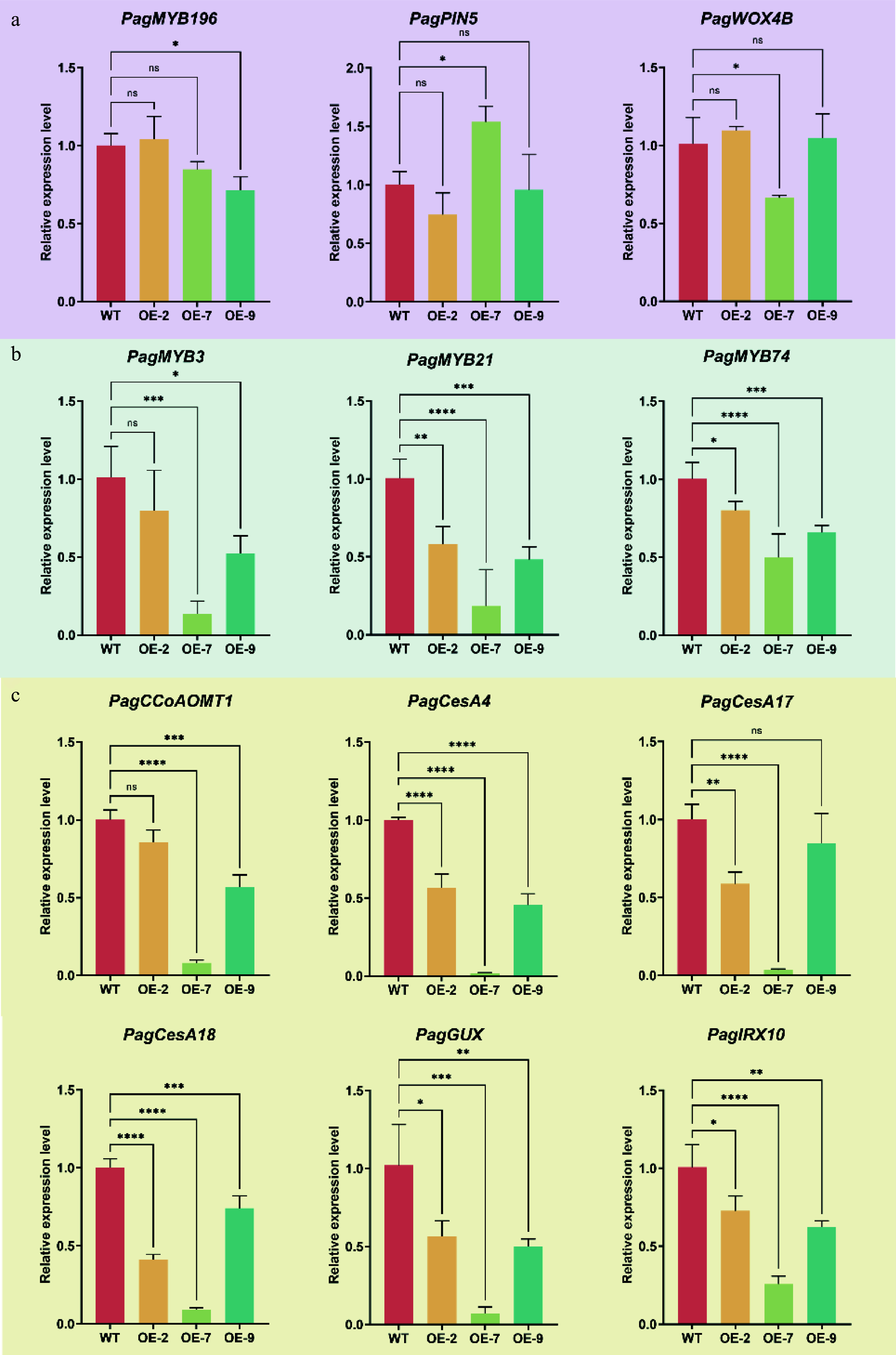
RT-qPCR analysis of key genes during wood formation in xylem tissues of WT and *PagTRM4B-a-OE* transgenic lines. (a) Relative expression levels of genes in cambial cell proliferation were determined by RT-qPCR. (b) Relative expression levels of transcription factors in SCW formation were determined by RT-qPCR. (c) Relative expression levels of SCW biosynthesis genes were determined by RT-qPCR. Error bars represent mean with SD. Asterisks indicate statistically significant differences between the WT and OE lines (one-way ANOVA followed by Dunnett's test for pairwise comparisons; * *p* < 0.05; ** *p* < 0.01; *** *p* < 0.001; **** *p* < 0.0001; n = 3 biological replicates, each with three technical replicates).

To explore PagTRM4B-a's regulatory role in wood formation, the expression of key marker genes across various developmental stages, such as cambial cell proliferation, xylem differentiation, and secondary cell wall biosynthesis were analyzed. RT-qPCR analysis indicated no significant difference in the expression levels of genes related to vascular cambium activity maintenance, including *PagMYB196*^[39]^, *PagPIN5*^[9]^, and *PagWOX4B*^[40]^ between WT and *PagTRM4B-a-OE* lines (Fig. 6a). In contrast, transcription factors involved in SCW formation, including *PagMYB3*^[5]^, *PagMYB21*^[41]^, and *PagMYB74*^[42]^, were significantly downregulated in the *PagTRM4B-a-OE* lines (Fig. 6b). Notably, genes associated with the biosynthesis of SCW components were significantly suppressed in *PagTRM4B-a-OE* lines (Fig. 6c). These included the lignin biosynthetic gene *PagCCoAOMT1*^[43]^, cellulose-related genes *PagCesA4*^[44,45]^, *PagCesA17*^[46]^, and *PagCesA18*^[47]^, as well as hemicellulose-associated genes such as *PagGUX*^[48]^ and *PagIRX10*^[49]^. These results indicate that PagTRM4B-a may modulate xylem differentiation and secondary cell wall biosynthesis through transcriptional regulation.

## Discussion

This study systematically characterized the TRM4 gene family in *P. alba* × *P. glandulosa*, revealing that PagTRM4B-a plays a crucial role in regulating secondary growth by modulating m^5^C RNA methylation and influencing downstream gene expression. While previous studies in Arabidopsis and rice have demonstrated that TRM4B orthologs are important for root development and stress responses^[28,29]^, the present findings extend the biological significance of TRM4B to wood formation in perennial woody plants.

Phylogenetic analysis and gene structure characterization confirmed that *PagTRM4B* belongs to the conserved NSUN family of RNA methyltransferase^[50−52]^. Expression profiling revealed that *PagTRM4B* maintains a relatively stable expression level in xylem tissues across developmental stages, suggesting its constitutive role in wood development rather than a stage-specific function. Elevated expression of *PagTRM4B-a* significantly altered tree architecture by reducing plant height, stem diameter, and internode number, suggesting a negative regulation of overall plant growth. Although the growth rate decreased, histological analysis of stem cross-sections showed a significant increase in secondary xylem width and cell layer number in OE lines, indicating improved xylem differentiation. This apparent paradox can be explained by the differential regulation of growth and secondary cell wall deposition. Slower primary growth may allow more resources or developmental time to be allocated to secondary growth, resulting in thicker xylem. In addition, the enhanced secondary wall deposition observed in *PagTRM4B-a-OE* plants may partially restrict longitudinal expansion, contributing to reduced internode elongation and overall plant height. Therefore, the increase in xylem width and the retardation of plant growth are not necessarily contradictory, but rather reflect a trade-off between primary and secondary growth rates.

Interestingly, the observed increase in xylem cell layers was not accompanied by significant changes in cambial width or cell layer number, indicating that PagTRM4B-a likely acts downstream of cambial cell proliferation to influence xylem maturation and/or secondary wall deposition. Consistently, wood compositional analysis revealed that *PagTRM4B-a* overexpression leads to increased lignin content but reduced cellulose and hemicellulose levels. Subsequent RT-qPCR analysis of several wood formation-related genes showed that the expression patterns of cambium-related genes, as well as cellulose and hemicellulose biosynthesis genes, were consistent with the observed phenotypes. Interestingly, only *PagCCoAOMT1* exhibited significantly decreased expression in *PagTRM4B-a-OE* plants, while the expression of other lignin biosynthesis-related genes was not significantly altered. Although a single lignin biosynthetic gene was downregulated, the overall lignin content in the transgenic poplar was increased. This phenomenon is not contradictory, as lignin biosynthesis is controlled by a highly branched and redundant metabolic network. Downregulation of a single structural gene does not necessarily reduce lignin accumulation; other paralogous genes or alternative pathways may compensate for the reduced activity of this gene, maintaining or even enhancing the metabolic flux toward lignin. Furthermore, ongoing RNA-seq and m5C-RNA-seq analyses will provide a more comprehensive understanding of the molecular mechanisms underlying this phenotype. These biochemical shifts align with the downregulation of key genes involved in secondary cell wall biosynthesis, supporting a broad regulatory role for PagTRM4B-a in secondary wall component accumulation.

As a putative m^5^C methyltransferase, PagTRM4B-a may exert these developmental effects through RNA modification. *PagTRM4B-a-OE* lines exhibited increased m^5^C levels in both total RNA and mRNA, suggesting PagTRM4B-a's role in regulating RNA methylation. Given the downregulation of SCW genes, it is hypothesized that overexpressed *PagTRM4B-a* alters m^5^C on mRNAs of these genes, leading to reduced transcript stability or translation, hence dampening xylem differentiation and cell wall biosynthesis. Moreover, considering that m^5^C has also been implicated in regulating alternative splicing and translation efficiency in plants, it is possible that PagTRM4B-a affects SCW gene expression through multiple layers of post-transcriptional regulation rather than stability alone. The absence of changes in cambial marker genes further supports the hypothesis that this mechanism acts at the differentiation stage, downstream of cambial proliferation. Key next steps will be essential to validate this proposed mechanism. Transcriptome-wide m^5^C mapping (e.g., m^5^C-RIP-seq) will help to identify direct targets of PagTRM4B-a. In parallel, experimental assays examining transcript stability and ribosome occupancy will help determine whether PagTRM4B-a primarily influences RNA fate through stability, translational efficiency, or splicing regulation. Such integrative analyses will be essential to establish a mechanistic framework linking m^5^C modification to wood formation.

## Conclusions

In this study, PagTRM4B-a was identified as an RNA m5C methyltransferase that is essential for xylem differentiation and SCW formation in *P. alba* × *P. glandulosa*. The present findings expand the epigenetic layer of regulation in wood development, highlighting the important role of RNA modifications in plant secondary growth. Future studies should further investigate the molecular mechanism by which PagTRM4B-a regulates wood formation. Taken together, this work provides mechanistic insights into PagTRM4B-a function and a potential target for improving wood traits in forestry species.

## SUPPLEMENTARY DATA

Supplementary data to this article can be found online.

## Data Availability

All data generated or analyzed during this study are included in this published article and its supplementary information files.
