# Supplementary material for: Identification of the RNA m5C methyltransferase genes in Populus alba × Populus glandulosa and the role of PagTRM4B in wood formation
Source: For Res (Fayettev). 2025 Nov 7;5:e025. doi: 10.48130/forres-0025-0025 (PMC12648020; doi:10.48130/forres-0025-0025)
Supplement: Supplementary file 1 — Supplementary data to this article can be found online. [file FR-2025-5-0025-Supplementary.zip › 10.48130_forres-0025-0025-Suppl-FigureS4.pdf]

a

b

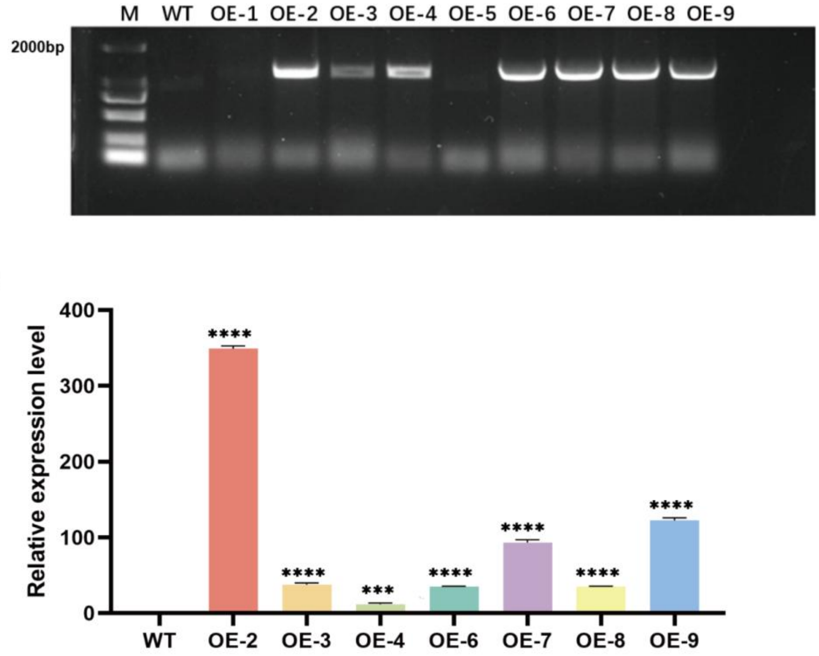

Fig.S4 Identification of *PagTRM4B-a-OE* transgenic plants. (a) PCR characterization of the *PagTRM4B-a-OE* transgenic plants. (b) RT-qPCR detection of the abundance of *PagTRM4B-a* in WT and *PagTRM4B-a-OE* transgenic plants. The expression level of *PagTRM4B-a* in WT was normalized to 1, and relative expression levels in transgenic lines were calculated accordingly. Error bars represent mean with SD. Asterisks indicate statistically significant differences between the WT and OE lines (one-way ANOVA followed by Dunnett's test for pairwise comparisons; *** $p < 0.001$； **** $p < 0.0001$; $n = 3$ biological replicates, each with 3 technical replicates).
