# Supplementary material for: Identification of the RNA m5C methyltransferase genes in Populus alba × Populus glandulosa and the role of PagTRM4B in wood formation
Source: For Res (Fayettev). 2025 Nov 7;5:e025. doi: 10.48130/forres-0025-0025 (PMC12648020; doi:10.48130/forres-0025-0025)
Supplement: Supplementary file 1 — Supplementary data to this article can be found online. [file FR-2025-5-0025-Supplementary.zip › 10.48130_forres-0025-0025-Suppl-TableS1.pdf]

Table S1. Primer sequences for gene clone and RT-qPCR.

Primer name	Primer Sequence (5'-3')
PagTRM4B-F	GGGACGAGATCGGTACCCGGGATGGGAGGGAGAGGAAGAGGA
PagTRM4B-R	GCTCACCATGTCGACTCTAGATCAAGCATCTGCAACCAGCTG
RT-PagTRM4B-F	AGGAAGAGGAAGCAGGTCAC
RT-PagTRM4B-R	AGGCGGGGTTTTGAGTAGAA
RT-PagTRM4C-F	AGAATCTGTGGAGCAAGCCT
RT-PagTRM4C-R	TTGCAGCATTCCGATTAGCC
RT-PagTRM4H-F	TCACCTTGCTGCCCTTATGA
RT-PagTRM4H-R	CAGTCCCAGATCCAGAGCAA
RT-PagMYB196-F	TGGGTGCATGTGGTAAAGAA
RT-PagMYB196-R	AAGGGGACTCCACTCCAAGT
RT-PagPIN5-F	GAACAGTGCGGGGCTATAAA
RT-PagPIN5-R	AAGGCAAGCACTGCCACTAT
RT-PagWOX4B-F	CACCCGGAAGGCAGATGA
RT-PagWOX4B-R	TAAAGAAAGAAAAGCACGTC
RT-PagMYB3-F	GGGCGATGGATTTTATGGCG
RT-PagMYB3-R	GAAGCAAGTGTTATCGAAGCAG
RT-PagMYB21-F	TCCATTTGCATTCCCTTCTC
RT-PagMYB21-R	CCTCCCATGACATCTTTGCT
RT-PagMYB74-F	TTTTGGGTAACAGGTGGGCT
RT-PagMYB74-R	CTTCTCCTCTTTCACCCCCAC
RT-PagCCoAOMT1-F	ATCTGCTGATGAAGGGCAAT
RT-PagCCoAOMT1-R	TTTCTGAATCACCGGGAGAC
RT-PagCesA4-F	CCAGGACTTGTGGCGTAATG
RT-PagCesA4-R	GAGGAGGGTGGTCCATTTGA
RT-PagCesA17-F	CCCCTCTAGTCACGGGCAACACAC
RT-PagCesA17-R	AAGGTGCACATTGAAGCACCATCG
RT-PagCesA18-F	GTTGGCCTCTGTCTTCTCTCTTGTT
RT-PagCesA18-R	CAATCAATGGAAATGCAGGTCTCCG
RT-PagGUX-F	CCTGACACTCCTAGAAAACGAC
RT-PagGUX-R	TGGACAGGATAAAACCCCTTG
RT-PagIRX10-F	GTGGGAAGGTTGAAGGTCTATG
RT-PagIRX10-R	CGAACTGGGCTGGATAAGAG
