# Supplementary material for: Identification of the RNA m5C methyltransferase genes in Populus alba × Populus glandulosa and the role of PagTRM4B in wood formation
Source: For Res (Fayettev). 2025 Nov 7;5:e025. doi: 10.48130/forres-0025-0025 (PMC12648020; doi:10.48130/forres-0025-0025)
Supplement: Supplementary file 1 — Supplementary data to this article can be found online. [file FR-2025-5-0025-Supplementary.zip › 10.48130_forres-0025-0025-Suppl-TableS2.pdf]

Table S2. Chromosomal location of *PagTRM4* genes and physicochemical properties of proteins

Gene Name	Gene ID	Chr	CDS length/bp	AA	MW	pI	GRAVY	Instability Idex
PagTRM4B-a	Pag.A07G002542.1	Chr07A	2508	835	93688.19	5.5	-0.493	40.41
PagTRM4B-b	Pag.B07G000528.1	Chr07B	2118	705	79494.07	5.7	-0.515	48.2
PagTRM4C-a	Pag.A01G004119.1	Chr01A	1965	654	73210.24	5.91	-0.797	53.21
PagTRM4C-b	Pag.B01G003873.1	Chr01B	2127	708	79055.42	5.92	-0.686	53.9
PagTRM4E-a	Pag.A01G003989.3	Chr01A	1731	576	64766.24	9.1	-0.23	47
PagTRM4E-b	Pag.B01G003858.3	Chr01B	1641	546	61411.46	9.4	-0.273	47.03
PagTRM4F-a	Pag.A05G002066.1	Chr05A	1758	585	64540.65	8.95	-0.327	37.27
PagTRM4F-b	Pag.B05G002042.1	Chr05B	1620	539	59373.31	6.9	-0.355	40.95
PagTRM4G-a	Pag.A07G002149.1	Chr07A	1185	394	43806.99	5.98	-0.21	37.87
PagTRM4G-b	Pag.B07G000997.2	Chr07B	1230	409	45702.23	6.1	-0.198	40.44
PagTRM4H-a	Pag.A06G000700.1	Chr06A	1560	519	57408.54	9.72	-0.292	43.45
PagTRM4H-b	Pag.B06G000601.1	Chr06B	1635	544	60369.16	9.68	-0.253	43.55
