# Supplementary material for: Identification of the RNA m5C methyltransferase genes in Populus alba × Populus glandulosa and the role of PagTRM4B in wood formation
Source: For Res (Fayettev). 2025 Nov 7;5:e025. doi: 10.48130/forres-0025-0025 (PMC12648020; doi:10.48130/forres-0025-0025)
Supplement: Supplementary file 1 — Supplementary data to this article can be found online. [file FR-2025-5-0025-Supplementary.zip › 10.48130_forres-0025-0025-Suppl-TableS3.pdf]

Table S3. The Ka/Ks ratios of duplication for *TRM4* genes between *P. alba* × *P. glandulosa* and *P. trichocarpa*.

Sequence	Ka	Ks	Ka/Ks
Pag.A01G003989.3-Potri.001G368500.1	0.0304912	0.0555868	0.548533
Pag.A01G004119.1-Potri.001G369600.3	0.0369608	0.0590809	0.625596
Pag.A05G002066.1-Potri.005G204900.4	0.0556567	0.0989822	0.56229
Pag.A06G000700.1-Potri.006G224000.1	0.013534	0.0443991	0.304827
Pag.A07G002149.1-Potri.007G056800.7	0.00896259	0.0252029	0.355617
Pag.A07G002542.1-Potri.007G096100.2	0.00617998	0.0276567	0.223453
Pag.B01G003858.3-Potri.001G368500.1	0.0514604	0.0807585	0.637213
Pag.B01G003873.1-Potri.001G369600.3	0.0257536	0.0732698	0.35149
Pag.B05G002042.1-Potri.005G204900.4	0.0158303	0.0351966	0.449767
Pag.B06G000601.1-Potri.006G224000.1	0.0143738	0.0501622	0.286546
Pag.B07G000528.3-Potri.007G096100.2	0.00450445	0.0454973	0.0990048
Pag.B07G000997.2-Potri.007G056800.7	0.00675143	0.0345007	0.19569
