# Supplementary material for: Identification of the RNA m5C methyltransferase genes in Populus alba × Populus glandulosa and the role of PagTRM4B in wood formation
Source: For Res (Fayettev). 2025 Nov 7;5:e025. doi: 10.48130/forres-0025-0025 (PMC12648020; doi:10.48130/forres-0025-0025)
Supplement: Supplementary file 1 — Supplementary data to this article can be found online. [file FR-2025-5-0025-Supplementary.zip › 10.48130_forres-0025-0025-Suppl-TableS4.pdf]

Table S4. The Ka/Ks ratios of duplication for *TRM4* genes between *P. alba* × *P. glandulosa* and *A. thaliana*.

Sequence	Ka	Ks	Ka/Ks
Pag.A01G003989.3-AT3G13180.1	0.174267	1.62615	0.107165
Pag.A01G004119.1-AT4G26600.1	0.292551	1.80691	0.161907
Pag.A01G004119.1-AT5G55920.1	0.282415	4.46059	0.0633135
Pag.A05G002066.1-AT1G06560.1	0.270984	2.17158	0.124787
Pag.A06G000700.1-AT5G26180.1	0.266295	1.55696	0.171036
Pag.A07G002149.1-AT5G66180.1	0.215721	4.2385	0.0508956
Pag.A07G002542.1-AT2G22400.1	0.258701	1.70902	0.151374
Pag.A07G002542.1-AT4G40000.1	0.282138	1.99794	0.141215
Pag.B01G003858.3-AT3G13180.1	0.184513	2.3409	0.0788212
Pag.B05G002042.1-AT1G06560.1	0.38061	3.20687	0.118686
Pag.B06G000601.1-AT5G26180.1	0.264823	1.66543	0.159011
Pag.B07G000528.3-AT2G22400.1	0.256679	1.71913	0.149307
Pag.B07G000528.3-AT4G40000.1	0.280708	2.14369	0.130946
Pag.B07G000997.2-AT5G66180.1	0.218079	4.19824	0.0519455
