# Supplementary material for: Identification of the RNA m5C methyltransferase genes in Populus alba × Populus glandulosa and the role of PagTRM4B in wood formation
Source: For Res (Fayettev). 2025 Nov 7;5:e025. doi: 10.48130/forres-0025-0025 (PMC12648020; doi:10.48130/forres-0025-0025)
Supplement: Supplementary file 1 — Supplementary data to this article can be found online. [file FR-2025-5-0025-Supplementary.zip › 10.48130_forres-0025-0025-Suppl-FigureS1.pdf]

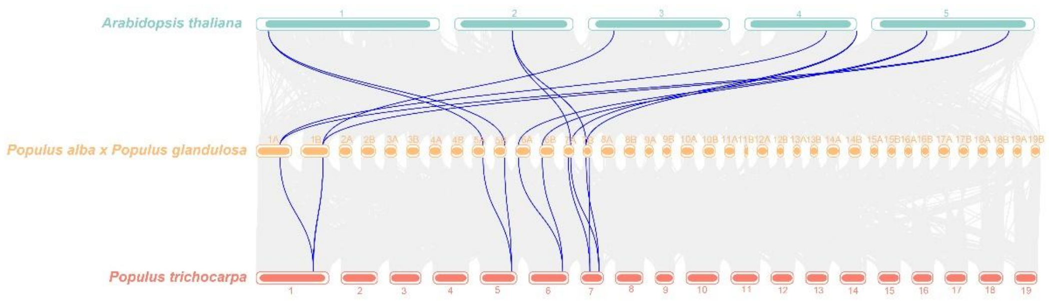

Fig.S1 Collinear analysis of *PagTRM4* genes in *P. alba* × *P. glandulosa* with *P. trichocarpa* and *A. thaliana*. Red boxes indicate chromosomes of *P. trichocarpa*, blue boxes indicate chromosomes of *A. thaliana*, yellow boxes indicate chromosomes of *P. alba* × *P. glandulosa*. Blue lines highlight the collinear *TRM4* gene pairs between *P. trichocarpa* and *P. alba* × *P. glandulosa* or *P. alba* × *P. glandulosa* and *A. thaliana*.
