# Supplementary figures and images for: Identification of the RNA m5C methyltransferase genes in Populus alba × Populus glandulosa and the role of PagTRM4B in wood formation
Source: For Res (Fayettev). 2025 Nov 7;5:e025. doi: 10.48130/forres-0025-0025 (PMC12648020; doi:10.48130/forres-0025-0025)

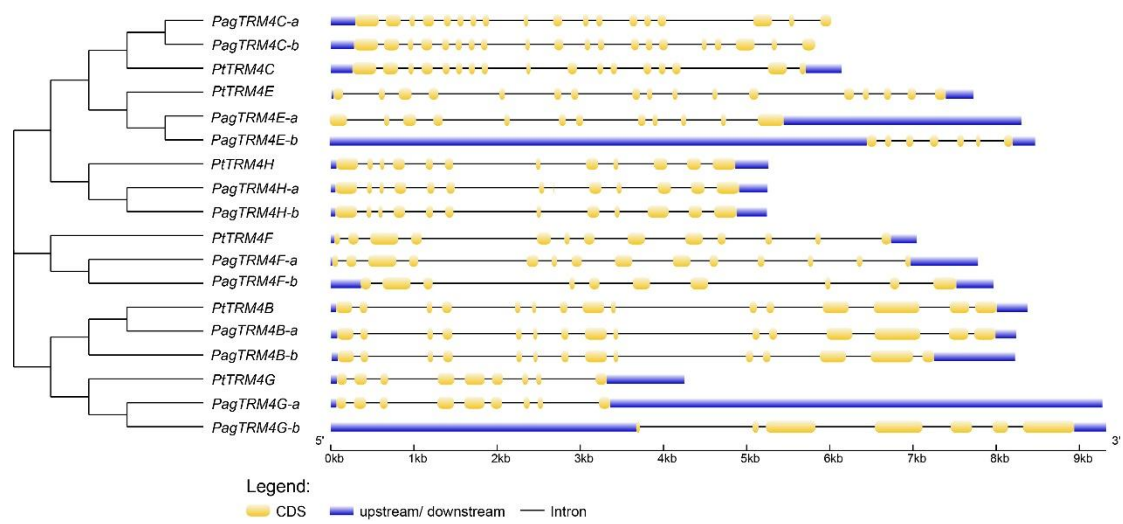

**Fig.S2** Phylogenetic relationships and gene structure of *PagTRM4* and *PtrTRM4* genes.

Supplement: Supplementary file 1 — Supplementary data to this article can be found online. [file FR-2025-5-0025-Supplementary.zip › 10.48130_forres-0025-0025-Suppl-FigureS2.pdf]
